# Supplementary material for: Cancer-independent somatic mutation of the wild-type NF1 allele in normal tissues in neurofibromatosis type 1
Source: Nat Genet. 2025 Feb 25;57(3):515–21. doi: 10.1038/s41588-025-02097-2 (PMC11906363; doi:10.1038/s41588-025-02097-2)
Supplement: Supplementary file 1 — Supplementary Note and Figs. 1–3. [file 41588_2025_2097_MOESM1_ESM.pdf]

nature genetics

Supplementary information https://doi.org/10.1038/s41588-025-02097-2

Cancer-independent somatic mutation of the wild-type *NF1* allele in normal tissues in neurofibromatosis type 1

In the format provided by the authors and unedited

SUPPLEMENTARY INFORMATION accompanying the manuscript 'Cancer-independent somatic mutation of the wild-type *NF1* allele in normal tissues in neurofibromatosis type 1'

TABLE OF CONTENTS

1. Supplementary note……………………………………………………………………. pp.1-9
2. Supplementary figures…………………………………………………………………. pp. 10-13
3. Supplementary references………………………………………………………… pp. 14-15

SUPPLEMENTARY NOTE

This note contains further details on the methods used in this study.

Assessment of DNA sequencing data quality

The CollectWgsMetrics tool from Picard (version 2.26.10) was used on the whole genome sequencing data to determine the median coverage and duplicate rate. The coverage cap was set to 500. CollectHsMetrics, also from Picard, was deployed for the whole exome sequencing data to determine the on-target coverage and duplicate rate.

Assessment of sample-to-sample concordance

Conpair (version 0.2)[1] was run between every sample that underwent whole genome or whole exome sequencing and a designated matched normal sample (PD50297b, PD51122q, PD51122aa3). Two samples were found to have >0.5% contamination and were excluded from the final data set analysed here (PD51122t_lo0011, PD51122t_lo0012). The remaining samples all showed a concordance of >99.5% with their matched normal.

Variant calling and filtering for whole genome and whole exome sequencing - substitutions

Substitutions were called using the CaVEMan algorithm (version 1.15.1)[2], unmatched against an *in silico* normal sample (PDv38is_wgs) for the whole genome data and matched (blood or skin) for the whole exome data. All called variants were initially filtered using soft flag filters contained within the VCF (ASRD ≥0.87 & CLPM = 0) to eliminate BWA-MEM-associated mapping errors. Further artefacts introduced as a result of cruciform DNA formation during DNA library preparation, a recognised consequence of the low input method used for microdissected tissue DNA in particular, were removed

using previously described filters[3]. Briefly, the artefacts generated by this phenomenon can be identified by their tendency to occur at similar alignment start positions on supporting reads.

The extended nucleotide contexts that passed variants were found in was examined. Some sequences derived from microdissected tissues showed an abundance of substitutions at loci with an upstream GGGTGGTCT, as has been previously noted[4]. Further hotspots were identified at loci with a preceding AGACCA and in regions of long (≥9bp) A or T repeats. Any substitution called where at least 8 of the upstream 9 bases matched GGGTGGTCT, or where the preceding 6 bases were AGACCA, or which occurred upstream or downstream of a poly-A or poly-T repeat ≥9bp long, was excluded. For the whole exome sequencing data, which was generated solely to expand our survey of the driver landscape, the only additional filter implemented was a cutoff of at least four supporting reads to consider a variant during driver curation.

Common SNPs were removed from the remaining unmatched whole genome sequencing by eliminating calls at loci reported to be mutated in gnomAD (version 3.1.1) with an allele frequency >0.1%. Recurrent artefacts identified within the Panel of Normals mask created by the Brain Somatic Mosaicism Network were also removed (PON.q20q20.05.5.fa). Bidirectional read support was required for passed substitutions to be considered further. At least one supporting read needed to be found in both directions and, where total locus coverage was >10, at least 15% of supporting reads needed to be found in both directions.

Genotyping of the unique, remaining mutations across all samples from a single case was performed using pileup (https://github.com/cancerit/vafCorrect, version 5.6.0), with defined mapping quality (MQ 30) and base quality (BQ 25) thresholds. Variants found at loci with a universally low coverage (≤10) across all case samples were removed, as were those which were not supported by at least four mutant reads in one or more samples. Examining the genotypes of variants across all non-CNS samples, where the risk of tumour infiltration was remote, remaining germline variants were excluded through the use of a one-sided exact binomial test. This test assumed that the fraction of reads supporting a germline mutation were drawn from a binomial distribution where $P = 0.5$, or 0.95 in the case of male sex chromosomes (null hypothesis). Somatic mutations should exist at a fraction beneath these cutoffs (alternative hypothesis). The Benjamini-Hochberg multiple hypothesis testing correction was applied to the resultant P values with a cutoff of $q < 10^{-5}$, as has been done before[5,6].

Certain artefacts, or germline mutations on DNA segments with constitutional copy number variation, may appear in multiple clonal samples at a low allele fraction. These calls were filtered by fitting a beta binomial distribution to the mutant read depth and total depth of each variant shared across two or more microdissected regions of epithelium and removing variants whose rho (overdispersion parameter) value was <0.1[5,6]. This was limited to epithelial samples as they should be tumour-free but sufficiently clonal to minimise the loss of true mosaic variants from within polyclonal tissues through their apparent underdispersion.

Substitutions that were identified across multiple cases and were not bonafide driver mutations likely represent recurrent artefacts, often as a result of mismapping. They were removed and only variants where >90% of reads at the locus had MQ >30 were kept. Any variants found within 5bp of an indel call within the unfiltered Pindel file (used for indel calling, see below) were also excluded as possible mapping artefacts. The final substitution filtering step involved calculating the background sequencing error rate for each mutation found in one of the three cases using the sequencing data from the other two cases, similar to the method employed by Shearwater[5-7]. Again, rare, shared driver events were not subject to this step. Variants that exceeded the error rate modelled from both unrelated bulk sequencing and microdissected samples were kept for subsequent analysis.

Variant calling and filtering for whole genome and whole exome sequencing - small insertions and deletions (indels)

For indels, the Pindel algorithm (cgpPindel version 3.5.0)[8] was used and 1bp indels at homopolymer runs of 9bp or longer were removed. As with the substitutions, the whole exome sequencing data required a minimum variant depth: this was set to five for indels. No further filtering was performed for the whole exome data before driver curation.

The same read direction, mean coverage, binomial test, beta binomial distribution and locus MQ (>90% MQ >30) filters were applied to the indels from the whole genome sequencing data as for the substitutions. The only notable differences were that a variant needed to have five or more supporting reads in at least one sample and the rho value cutoff was set to 0.2. For indels, genotyping was carried out using Exonerate (https://github.com/cancerit/vafCorrect, version 5.6.0) which may annotate reads as 'ambiguous' or 'unknown' if they cannot be assigned to wild-type or the interrogated mutant allele. If >10% of reads at a locus were assigned to these categories, the variant was discarded.

Variant calling and filtering for whole genome and whole exome sequencing - copy number changes

Somatic copy number variants in the whole genome data were identified by two different callers. The first, Battenberg (cgpBattenberg version 3.5.3)[9], was run using the bulk data only with bulk blood or skin samples acting as the matched normal sample. The second, ASCAT (AscatNGS version 4.3.2)[10], was applied to all samples using the same matched normal samples and a segmentation penalty of 100. To eliminate technical sources of variation in coverage that add noise to the logR profile, ascatPCA was applied (https://github.com/hj6-sanger/ascatPCA). This performs a principal component analysis using a panel of unrelated, normal samples to define recurrent regions of artefact change and uses these to eliminate false positive calls[11]. The quality of the resultant copy number calls could be assessed with a 'goodness of fit' metric. Furthermore, orthogonal validation was also possible in the samples with a high substitution burden, especially if they had a high tumour purity. This is because the VAF of substitutions on a segment must be concordant with its copy number state and the purity of the sample they are found in. To this end, we ran CNAqc (version 1.0.0)[12] using the substitution calls and the ascatPCA copy number calls and purity estimates. Using the original tumour purity estimate from ascatPCA, we found that only 30% (26/88) of PD51122 sample copy number calls with ≥40% tumour purity passed the CNAqc check using default parameters, compared to 92% (48/52) and 89% (34/38) of PD50297 and PD51123 tumour samples respectively. On closer inspection, whilst the purity estimates between ascatPCA and our method of estimating tumour purity (see 'Estimating Tumour Infiltration' below) were very similar for the near-diploid tumours, ascatPCA provided higher than expected purity estimates for the aneuploid PD51122 (**Supplementary Figure 1a**). Our method of estimating purity showed good concordance with Battenberg calls in all tumours for the bulk samples (**Supplementary Figure 1b**) so we ran CNAqc again for the PD51122 tumour samples with the purity from our method. This markedly improved the concordance between substitutions and copy number calls with 76% passing (67/88), indicating our method of inferring purity better accounted for the copy number calls and substitution VAFs. In Supplementary table 3, the 'PASS/FAIL' CNAqc column uses the original ascatPCA purity except for PD51122 where the ≥40% tumour purity samples use the purity derived from our approach. We kept copy number data from all samples where the ascatPCA goodness of fit was ≥95% and tumour purity (our method) <40%. For samples with ≥40% tumour purity (our method), we kept calls if the average number of reads per chromosome copy was ≥5 and either ascatPCA goodness of fit was ≥95% or the sample passed CNAqc.

Variant calling and filtering for whole genome and whole exome sequencing - structural variants

GRIDSS (version 2.9.4)[13] was run to call structural variants (SVs), using default settings. Only SVs larger than 1kb were considered. SVs 1-30 kb in size needed quality scores ≥300 but those larger than 30 kb used a cutoff of 250. SVs required breakpoint assembly on both sides with at least four discordant and

two split reads supporting them. Where breakpoints were imprecise, defined as the start and end positions being >10bp apart, the SV was filtered out. SVs where the standard deviation of the alignment position at the ends of discordant read pairs was <5 were also removed. Lastly, to remove potential germline SVs and recurrent artefacts, we removed SVs found in at least three different samples from a panel of normal samples or in the matched normal sample[14].

Variant calling and filtering for duplex sequencing of the *NF1* gene

For each patient, a matched normal was created by aggregating samples from the same patient. Patients PD50297, PD51122, and PD51123 had sufficient samples contributing to their matched normals that germline mutations could be confidently removed and no hard VAF cutoff was applied. Samples from the validation cohort had fewer tissues per donor, and so any mutation at an allele fraction of greater than 0.1 in the deduplicated bam was excluded on the grounds that it may be a germline variant.

Reads were collapsed into read bundles that originate from the same duplex DNA molecule using their adaptor sequences. Mutations were called if they were supported by reads from both strands and were not called in the normal sample[15,16].

Duplex sequencing is vulnerable to trace amounts of contamination as a result of its ability to detect mutations on a single molecule of DNA. Contaminating DNA may be either human or non-human. In order to filter out non-human contamination when calling substitutions, only read bundles that had either 0 or 1 single-stranded or double-stranded mismatches relative to the reference genome in addition to the called substitution were included in the analysis. This filters out contamination with DNA from other species, which may have multiple mismatches per read bundle, as well as reads with large amounts of single-stranded DNA damage. Contamination with human DNA may be either from samples that are on the same plate (either biological contamination through errant DNA molecules or bioinformatic contamination through index hopping), or from DNA that is not on the plate. To remove contaminants from the same plate, every sample on the plate was genotyped both with GATK HaplotypeCaller[17] (version 4.2.4.1) and BCFTools[18] (version 1.9), and no mutation that was called as germline in any sample was considered for a given plate. Second, any mutation catalogued as germline in Gnomad v2[19] with an allele fraction of greater than 0.1% was removed from the analysis. After these steps, the global dN/dS value for the dataset was 0.93 (95% CI 0.85-1.03). Reassuringly, contamination with other species or germline DNA from other humans contains an excess of synonymous mutations and artificially lowers the dN/dS ratio. Any contamination that passes our filters will reduce our ability

to detect positive selection, but is highly unlikely to contribute to detection of positive selection where there is none.

After filtering, mutations were analysed using the package dNdScv[20] (version 0.0.1.0). This tool uses a maximum likelihood method to quantify selection, integrating genomic covariates to correct for mutation biases. No limit was applied to the number of mutations with the *NF1* gene or the number of mutations per sample, and the duplex sequencing coverage of *NF1* in the group being tested was used as an offset.

One variant in *NF1*, chr 17:31226459 A>AC resulting in p.I679fs, was called by duplex sequencing in five separate tissues from PD51122 at low allele fraction: the pons (VAF 0.02214), the medulla (VAF 0.00744), the occipital cortex (VAF 0.00096), the small bowel (VAF 0.00147), and a spindle cell lesion of the ankle (VAF 0.00059). It was also previously found by whole genome sequencing of the pons and genotyped in the medulla. Four possible explanations for this recurrence presented themselves:

(i) The variant was an artefact.
(ii) The variant was found in one tissue that contaminated other tissues ('carryover').
(iii) The variant was embryonic, i.e. occurred in a common ancestor of cells in all of these tissues.
(iv) The variant was acquired independently, multiple times.

To address each in turn:

(i) The variant was called by whole genome sequencing and duplex sequencing. The latter has an extremely low error rate. The variant has only been found in the patient with neurofibromatosis type 1, and so is unlikely to represent a mapping error or similar. All occurrences of this variant are also out of phase with the germline *NF1* variant. It is therefore unlikely to be an artefact.

(ii) If material from one tissue was contaminating another, numerous mutations from the source tissue should be found in the contaminated tissue rather than just the *NF1* variant. Additionally, we would not expect to find carryover mutations in laser capture microdissected biopsies, since this tissue has been directly visualised and stray cells would be apparent. Assuming that the pons is the source of the mutation (as the mutation was found here by whole genome sequencing, and the VAF is highest here), we can look for pontine variants in other tissues. Looking in the medulla, for instance, of 110 pontine substitutions, 14 were found in the medulla. Of these, 11 were present in laser capture microdissections of the medulla. Only 3 mutations, therefore, are consistent with carryover. This is an implausibly low number to suggest carryover.

(iii) It is highly unlikely that a genuine embryonic variant that has occurred across developmentally distantly related organs such as the bowel and the central nervous system would be at such low allele fraction in these different tissues. Such a finding would imply a cell that existed late in development that could give rise to both bowel and brain. For the mutation that we identified across more than one germ layer in whole genome sequencing data (Y489C), our analysis of other shared mutations (**Fig. 3e**) indicated that they were independent events and not embryonic. Furthermore, the spindle cell lesion of the ankle is clonal. An embryonic variant within the neoplasm, therefore, should be clonal within it, and not at the low VAF that we observe.

(iv) On first inspection, the same precise DNA changes are unlikely to occur multiple times. The mutation, however, involves slippage in a cytosine homopolymer, which may be highly mutable. Exactly the same mutation has occurred in 60 different cancers as documented in Cosmic[21], such that this locus has far more indels than any other in the *NF1*. The high dN/dS values associated with *NF1* truncating mutations on a background of neurofibromatosis type 1 suggest that essentially every time a truncating variant occurs in *NF1* it is likely to be selected for, and so we have a good chance of finding it.

A combination of the above explanations is also possible. For example, the mutation may be a shared embryonic variant between the pons and medulla, but has occurred again independently in the ankle lesion. Following the reasoning above, we considered independent mutations to be most likely and the results presented in the main text treat them as such. The dN/dS analysis quantifying selection pressures in *NF1* has been repeated both treating the mutations as independent and only allowing each variant to be counted only a single time in each patient. The conclusions drawn from the data remain the same using both methods (**Supplementary Figure 2**).

Tumour phylogeny reconstruction

Phylogenies were created from the bulk tumour samples with purity ≥40% only, to ensure confidence in the copy number calling estimates. Battenberg copy number calls were used to generate cancer cell fractions (CCFs) for the substitutions which were then subject to multidimensional DPClust (ndDPClust, implemented using DPClust version 2.2.2)[5,22]. The Gibbs sampler was run for 1,000 iterations with 200 dropped as burn-in per tumour. The resultant mutation clusters were then inspected and kept, split or merged depending on the distribution of the CCFs of their substitutions across all samples. Only clusters containing at least 1% of substitutions were kept (**Supplementary table 9**). The final clusters demonstrated the clonal trunk and multiple subclones found across each tumour.

Estimating tumour infiltration

The truncal mutations (both driver and passenger mutations) identified during tree building provided a set of variants that would be expected in any sample infiltrated by tumour. All samples from each patient, irrespective of their distance from the tumour, were examined for evidence of tumour infiltration. For the two near-diploid tumours (PD50297 and PD51123), we identified any truncal variant found at a site with one clonal copy of each allele in all bulk tumour samples with a purity ≥40%, resulting in 384 and 259 clonal substitutions respectively. The mean VAF, excluding outliers, of these variants was calculated and doubled to provide an estimate for tumour infiltration. Even a normal sample that contains no tumour cells may share early embryonic variants with a tumour, and it is important to distinguish embryonic variants from truncal tumour variants; this may be done based on the overall distribution of the VAFs in the sample. A normal sample with no tumour infiltration will share a handful of high VAF variants with the tumour (representing embryonic variants shared by the tumour and normal tissue) whilst a sample with low level tumour involvement will possess many truncal variants at low VAFs. To remove outlier embryonic variants, VAF vectors were trimmed to only include variants whose VAF in a given sample was no further than 1.5× interquartile range below the lower or above the upper quartile. Any variant outside of this range was deemed an outlier. For the grossly aneuploid tumour (PD51122), the substitutions at uniformly clonal 2+0 copy number sites that were estimated to be found on both copies of the allele were used to estimate tumour infiltration; no doubling of the VAF was required. PD51122 had 52 substitutions that fulfilled these criteria.

This approach was evaluated using two methods. First, for the bulk tumour samples with a purity ≥10%, the minimum threshold typically required for copy number callers, we could compare our purity estimates with those generated by Battenberg. There was a near perfect, positive correlation in the results between the two methods (**Supplementary Figure 1b**). Second, to establish the efficacy of our method for samples with <10% tumour involvement, we simulated data to capture a wide range of trunk lengths, sample coverage and tumour involvement. For each simulation (1,000 in total per scenario), the total coverage for each locus in the tumour trunk was randomly sampled from a Poisson distribution with a mean equal to the average sample coverage. The variant depth was generated from a binomial distribution whose number of trials was equal to the coverage generated by the aforementioned Poisson distribution and probability of success was half the purity, like the scenario used for the near-diploid tumours. The fraction of simulations, for a given number of truncal mutations, coverage and tumour purity, that returned a tumour infiltrate value >0, provided a probability for detecting tumour, if there was genuine tumour involvement in a sample. In the worst

case scenario, with only 50 substitutions to use in the pile-up, we estimated we had >95% chance to detect 3% tumour infiltration and 100% chance at 5% infiltration using our approach for a sample with 30× coverage (**Supplementary Figure 3**).

For the whole exomes, where the baitset would not capture most truncal mutations identified in the whole genomes, the tumour purity was approximated using the variant allele fraction of each tumour's *TP53* hotspot mutation, correcting for its copy number state.

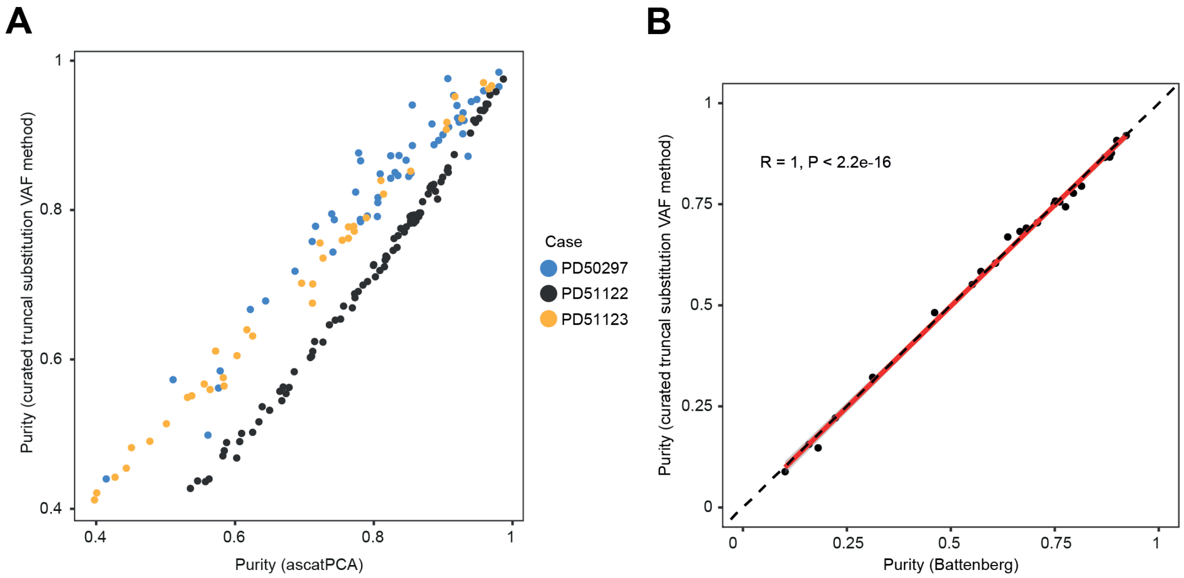

Supplementary Figure 1. Comparison of tumour purity estimates between three methods. a, Our truncal mutation VAF method versus ascatPCA, restricted to tumour samples with a tumour purity ≥40% (as determined by the former method). **b**, Our method versus Battenberg, restricted to bulk tumour samples with a tumour purity ≥10% (as determined by Battenberg). A Pearson correlation between the two axes is shown on the graph, with the *P* value determined by a two-sided test. A linear regression line is fit to the data (red, solid), with 95% confidence intervals (grey ribbon). It closely matches a line representing the equation y = x (black, dashed).

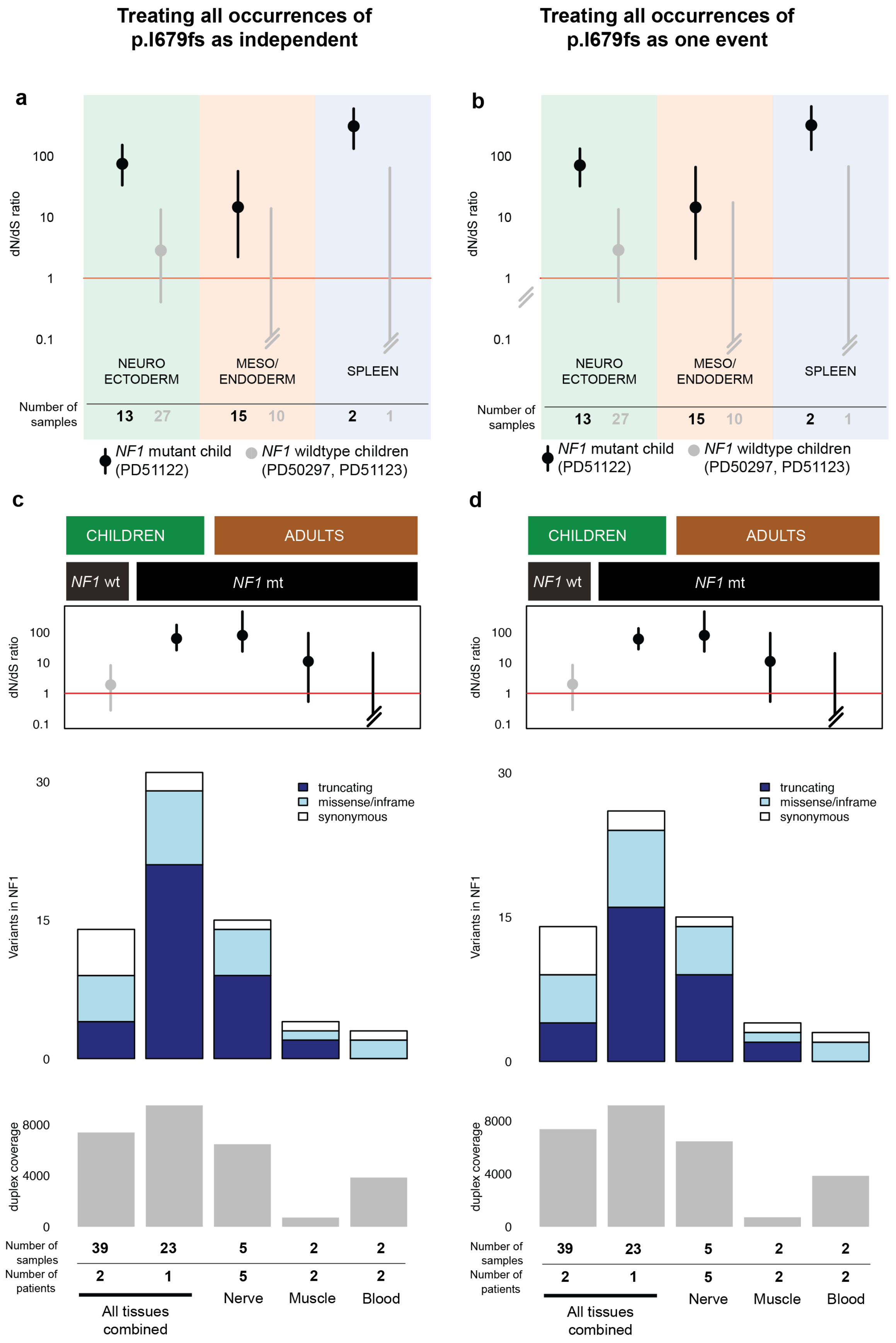

Supplementary Figure 2. Duplex sequencing data analysed considering the same mutation as independent or as the same event. One variant in *NF1*, chr 17:31226459 A>AC resulting in p.I679fs,

was called by duplex sequencing in five separate tissues from PD51122. These may have occurred as independent mutations or may represent the same event (**Supplementary Note**). Here, the results are shown comparing each occurrence of this mutation as independent events (panels a and c; as in Fig. 3f and g) or as the same event (panels b and d). a and b, dN/dS ratios for truncating variants, according to germ layer and *NF1* germline mutation status. The dot represents the maximum likelihood estimate and the black lines represent the 95% credible interval. When the lower bound of the credible interval is above 1 (red line), there is statistically significant positive selection. c and d, normal tissues from adults with neurofibromatosis type 1 are grouped by tissue type and evaluated for an excess of non-synonymous variants in *NF1*, and compared with the three index children. Upper, dN/dS ratios for truncating mutations; middle, counts of variants in *NF1*; lower, total duplex coverage (**Methods**) over *NF1* in each group. In a, b, c, and d, if the credibility interval extends beyond the range of the plot it is shown as truncated with two slanted lines.

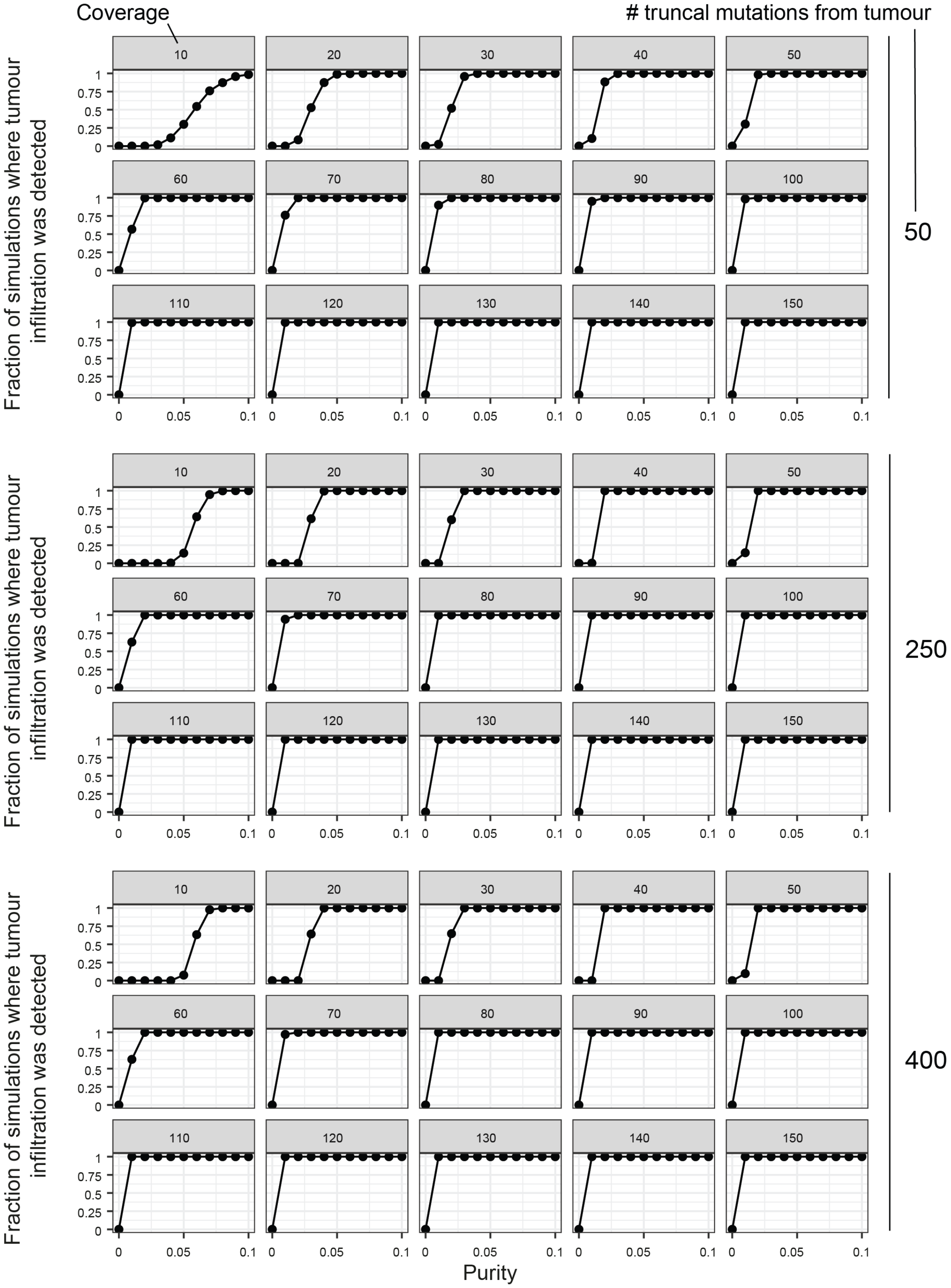

Supplementary Figure 3. Simulated data estimating the efficacy of our approach to detect low level tumour infiltration. The range of truncal mutation catalogue sizes used reflects the variability seen in our cohort.

301

SUPPLEMENTARY REFERENCES

1. Bergmann E.A. et al. Conpair: concordance and contamination estimator for matched tumor-normal pairs. Bioinformatics 32, 3196-8 (2016)
2. Jones D, Raine KM, Davies H, et al. cgpCaVEManWrapper: Simple Execution of CaVEMan in Order to Detect Somatic Single Nucleotide Variants in NGS Data. Curr Protoc Bioinformatics 56, 1501-08 (2016)
3. Ellis, P. et al. Reliable detection of somatic mutations in solid tissues by laser-capture microdissection and low-input DNA sequencing. Nat. Protoc. 16, 841–871 (2021)
4. Robinson, P.S. et al. Increased somatic mutation burdens in normal human cells due to defective DNA polymerases. Nat Genet 53, 1434-1442 (2021)
5. Oliver TRW, Chappell L, Sanghvi R, et al. Clonal diversification and histogenesis of malignant germ cell tumours. Nat Commun. 13, 4272 (2022)
6. Coorens THH, Treger TD, Al-Saadi R, et al. Embryonal precursors of Wilms tumor. Science. 366, 1247-1251 (2019)
7. Martincorena, I. et al. Tumor evolution. High burden and pervasive positive selection of somatic mutations in normal human skin. Science 348, 880–886 (2015)
8. Ye K, et al. Pindel: a pattern growth approach to detect break points of large deletions and medium sized insertions from paired-end short reads. 25:2865-71 (2009)
9. Nik-Zainal S, Van Loo P, Wedge DC, et al. The life history of 21 breast cancers. Cell, 149, 994-1007 (2012)
10. Raine KM, Van Loo P, Wedge DC, et al. ascatNgs: Identifying Somatically Acquired Copy-Number Alterations from Whole-Genome Sequencing Data. Curr Protoc Bioinformatics 56,1591- 97 (2016)
11. Robinson PS, Thomas LE, Abascal F, et al. Inherited MUTYH mutations cause elevated somatic mutation rates and distinctive mutational signatures in normal human cells. Nat Commun 13, 3949 (2022)
12. Househam J, Bergamin R, Milite S, et al. Integrated quality control of allele-specific copy numbers, mutations and tumour purity from cancer whole genome sequencing assays. bioRxiv 2021.02.13.429885.
13. Cameron DL, Schroder J, Penington JS, et al. GRIDSS: sensitive and specific genomic rearrangement detection using positional de Bruijn graph assembly. Genome Res, 27, 2050-60 (2017)
14. Mitchell E, Spencer Chapman M, Williams N, et al. Clonal dynamics of haematopoiesis across the human lifespan. Nature 606, 343-50 (2022)
15. Abascal F, et al. Somatic mutation landscapes at single-molecule resolution. Nature. 593, 405–410 (2021)
16. Lawson, A.R.J, Abascal F., Nicola P.A. et al, et al. Somatic mutation and selection at epidemiological scale. Preprint at https://www.medrxiv.org/content/10.1101/2024.10.30.24316422v1.full (2024)
17. Van der Auwera GA & O'Connor BD. Genomics in the Cloud: Using Docker, GATK, and WDL in Terra (1st Edition). (O'Reilly Media, 2020)
18. Li H. A statistical framework for SNP calling, mutation discovery, association mapping and population genetical parameter estimation from sequencing data. Bioinformatics 27(21) 2987-93 (2011)
19. Karczewski K. et al. The mutational constraint spectrum quantified from variation in 141,456 humans. Nature 581, 434-443 (2020)
20. Martincorena, I. et al. Universal patterns of selection in cancer and somatic tissues. Cell 171, 1029–1041 (2017)
21. Tate et al. COSMIC: the Catalogue Of Somatic Mutations In Cancer. Nucleic Acids Res 8;47 (2019)

22. Rabbie R, Ansari-Pour N, Cast O, et al. Multi-site clonality analysis uncovers pervasive heterogeneity across melanoma metastases. Nat Commun 11, 4306 (2020)
