# Supplementary material for: Cancer-independent somatic mutation of the wild-type NF1 allele in normal tissues in neurofibromatosis type 1
Source: Nat Genet. 2025 Feb 25;57(3):515–21. doi: 10.1038/s41588-025-02097-2 (PMC11906363; doi:10.1038/s41588-025-02097-2)
Supplement: Supplementary file 2 — Reporting Summary [file 41588_2025_2097_MOESM2_ESM.pdf]

nature portfolio

Corresponding author(s): Sam Behjati
Thomas Jaques
Inigo Martincorena
Kieren Allinson

Last updated by author(s): Nov 22, 2024

# Reporting Summary

Nature Portfolio wishes to improve the reproducibility of the work that we publish. This form provides structure for consistency and transparency in reporting. For further information on Nature Portfolio policies, see our Editorial Policies and the Editorial Policy Checklist.

## Statistics

For all statistical analyses, confirm that the following items are present in the figure legend, table legend, main text, or Methods section.

| n/a | Confirmed | |
|---|---|---|
| ☐ | ☒ | The exact sample size (*n*) for each experimental group/condition, given as a discrete number and unit of measurement |
| ☐ | ☒ | A statement on whether measurements were taken from distinct samples or whether the same sample was measured repeatedly |
| ☐ | ☒ | The statistical test(s) used AND whether they are one- or two-sided<br>*Only common tests should be described solely by name; describe more complex techniques in the Methods section.* |
| ☐ | ☒ | A description of all covariates tested |
| ☐ | ☒ | A description of any assumptions or corrections, such as tests of normality and adjustment for multiple comparisons |
| ☐ | ☒ | A full description of the statistical parameters including central tendency (e.g. means) or other basic estimates (e.g. regression coefficient) AND variation (e.g. standard deviation) or associated estimates of uncertainty (e.g. confidence intervals) |
| ☐ | ☒ | For null hypothesis testing, the test statistic (e.g. *F*, *t*, *r*) with confidence intervals, effect sizes, degrees of freedom and *P* value noted<br>*Give P values as exact values whenever suitable.* |
| ☐ | ☒ | For Bayesian analysis, information on the choice of priors and Markov chain Monte Carlo settings |
| ☒ | ☐ | For hierarchical and complex designs, identification of the appropriate level for tests and full reporting of outcomes |
| ☐ | ☒ | Estimates of effect sizes (e.g. Cohen's *d*, Pearson's *r*), indicating how they were calculated |

*Our web collection on statistics for biologists contains articles on many of the points above.*

## Software and code

Policy information about availability of computer code

| | |
|---|---|
| Data collection | No software was used for data collection. |
| Data analysis | Publicly available code is referenced in the manuscript (please see Methods and Supplementary Note). Software versions used were: Picard (version 2.26.10) ,Conpair (version 0.2), CaVEMan algorithm (version 1.15.1), vafCorrect (https://github.com/cancerit/vafCorrect, version 5.6.0), cgpPindel version 3.5.0, Battenberg (cgpBattenberg version 3.5.3), ascatPCA (https://github.com/hj6-sanger/ascatPCA), CNAqc (version 1.0.0), GRIDSS (version 2.9.4), GATK HaplotypeCaller (version 4.2.4.1), BCFTools (version 1.9), dNdScv (version 0.0.1.0), DPClust (version 2.2.2)<br><br>Custom R code for bespoke analyses is available at https://github.com/trwo/nf1_second_hit_normal_tissues. |

For manuscripts utilizing custom algorithms or software that are central to the research but not yet described in published literature, software must be made available to editors and reviewers. We strongly encourage code deposition in a community repository (e.g. GitHub). See the Nature Portfolio guidelines for submitting code & software for further information.

## Data

Policy information about availability of data

All manuscripts must include a data availability statement. This statement should provide the following information, where applicable:

- Accession codes, unique identifiers, or web links for publicly available datasets
- A description of any restrictions on data availability
- For clinical datasets or third party data, please ensure that the statement adheres to our policy

Whole-genome and targeted sequencing data are being deposited in the European Genome-Phenome Archive (EGA; https://www.ebi.ac.uk/ega/), with accession number with accession ID EGAD00001015398. Mutation calls are available as supplementary tables or as a supplementary dataset on Mendeley data (https://doi.org/10.17632/hfv45sg3c5.1). Datasets used in the analyses include: gnomAD (version 3.1.1), COSMIC (version 94), and GRCh38 human reference genome.

## Research involving human participants, their data, or biological material

Policy information about studies with human participants or human data. See also policy information about sex, gender (identity/presentation), and sexual orientation and race, ethnicity and racism.

| | |
|---|---|
| Reporting on sex and gender | Sex and gender were not considered in the design of the study, and are not reported in the analyses. |
| Reporting on race, ethnicity, or other socially relevant groupings | Race, ethnicity, and other social groupings were not considered in the design of the study, and are not reported in the analyses. |
| Population characteristics | As children who die of brain cancer are - fortunately - rare, and those with neurofibromatosis type 1 are a subset of those, care has been taken to preserve their anonymity. Age ranges have been provided rather than precise ages for this cohort, and the sex has not been provided, deliberately. For the same reason, the ethics of the study that recruited the adult cohort do not allow us to divulge precise patient characteristics that could allow patient identification. We therefore only identify them as adults with neurofibromatosis type 1. We studied all cases of children who died of brain tumours who had consented to post mortem studies in which tissue could be used for research purposes at our collaborating centres within the study period (of approximately two years). Of these, only one had neurofibromatosis type 1. For our validation cohort, all cases of patients with neurofibromatosis type 1 and tissue available at our collaborating centre were studied. |
| Recruitment | Please see details in the text and methods. |
| Ethics oversight | Study of the discovery cohort of three children was approved by NHS research ethics committees (PD50297 - HRA East Midlands Derby REC, 08/H0405/22+5; PD51122 & PD51123 - London Brent REC, 16/LO/0960). Study of the validation cohort of adults with neurofibromatosis type 1 was approved by NHS research ethics committees (20/YH/0088, IRAS 272816, NHS Yorkshire & The Humber - Leeds East Research Ethics Committee). |

Note that full information on the approval of the study protocol must also be provided in the manuscript.

# Field-specific reporting

Please select the one below that is the best fit for your research. If you are not sure, read the appropriate sections before making your selection.

☒ Life sciences ☐ Behavioural & social sciences ☐ Ecological, evolutionary & environmental sciences

For a reference copy of the document with all sections, see nature.com/documents/nr-reporting-summary-flat.pdf

# Life sciences study design

All studies must disclose on these points even when the disclosure is negative.

| | |
|---|---|
| Sample size | The study was designed in two phases. In the first phase, a discovery cohort of three children, of whom one had neurofibromatosis type 1 and two did not, was investigated. In the second phase, a validation cohort of ten adults, all of whom had neurofibromatosis type 1, was investigated. The sample size in each case was determined by tissue availability. No statistical method was used to determine sample size. Fortunately, only a small number of children die of brain tumours. We studied all cases of children who died of brain tumours who had consented to post mortem studies in which tissue could be used for research purposes at our collaborating centres within the study period (of approximately two years). Of these, only one had neurofibromatosis type 1. For our validation cohort, all cases of patients with neurofibromatosis type 1 and tissue available at our collaborating centre were studied. The experiments were not randomized and the investigators were not blinded to whether patients had neurofibromatosis type 1 during experiments and outcome assessment. |
| Data exclusions | One patient from the validation cohort was excluded as no NF1 germline variant was identified. |
| Replication | Multiple sequencing modalities were used (WGS, WES, duplex sequencing) to validate the detection of mutations. Each individual experiment was not replicated, as each experiment uses up the tissue that has been investigated in the experiment itself. |

| | |
|---|---|
| Randomization | No randomization was carried out. Covariates were not controlled. This is not important in our study as our study is an exploratory analysis of the somatic mutation landscape in a very rare situation of a child with neurofibromatosis type 1 undergoing a post mortem examination. |
| Blinding | The study was not blinded. No conditions were tested, and so blinding would not be meaningful. |

# Reporting for specific materials, systems and methods

We require information from authors about some types of materials, experimental systems and methods used in many studies. Here, indicate whether each material, system or method listed is relevant to your study. If you are not sure if a list item applies to your research, read the appropriate section before selecting a response.

## Materials & experimental systems

| n/a | Involved in the study |
|---|---|
| ☒ | ☐ Antibodies |
| ☒ | ☐ Eukaryotic cell lines |
| ☒ | ☐ Palaeontology and archaeology |
| ☒ | ☐ Animals and other organisms |
| ☒ | ☐ Clinical data |
| ☒ | ☐ Dual use research of concern |
| ☒ | ☐ Plants |

## Methods

| n/a | Involved in the study |
|---|---|
| ☒ | ☐ ChIP-seq |
| ☒ | ☐ Flow cytometry |
| ☒ | ☐ MRI-based neuroimaging |

## Plants

| | |
|---|---|
| Seed stocks | *Report on the source of all seed stocks or other plant material used. If applicable, state the seed stock centre and catalogue number. If plant specimens were collected from the field, describe the collection location, date and sampling procedures.* |
| Novel plant genotypes | *Describe the methods by which all novel plant genotypes were produced. This includes those generated by transgenic approaches, gene editing, chemical/radiation-based mutagenesis and hybridization. For transgenic lines, describe the transformation method, the number of independent lines analyzed and the generation upon which experiments were performed. For gene-edited lines, describe the editor used, the endogenous sequence targeted for editing, the targeting guide RNA sequence (if applicable) and how the editor was applied.* |
| Authentication | *Describe any authentication procedures for each seed stock used or novel genotype generated. Describe any experiments used to assess the effect of a mutation and, where applicable, how potential secondary effects (e.g. second site T-DNA insertions, mosiacism, off-target gene editing) were examined.* |
